# The Involvement of microRNAs in Plant Lignan Biosynthesis—Current View

**DOI:** 10.3390/cells11142151

**Published:** 2022-07-08

**Authors:** Katarína Ražná, Ľubomír Harenčár, Matúš Kučka

**Affiliations:** Institute of Plant and Environmental Sciences, Faculty of Agrobiology and Food Resources, Slovak University of Agriculture in Nitra, Tr. A. Hlinku 2, 94976 Nitra, Slovakia; xharencar@uniag.sk (Ľ.H.); xkucka@uniag.sk (M.K.)

**Keywords:** secondary metabolites, phenylpropanoids, lignans, microRNAs, transcription factors

## Abstract

Lignans, as secondary metabolites synthesized within a phenylpropanoid pathway, play various roles in plants, including their involvement in growth and plant defense processes. The health and nutritional benefits of lignans are unquestionable, and many studies have been devoted to these attributes. Although the regulatory role of miRNAs in the biosynthesis of secondary metabolites has been widely reported, there is no systematic review available on the miRNA-based regulatory mechanism of lignans biosynthesis. However, the genetic background of lignan biosynthesis in plants is well characterized. We attempted to put together a regulatory mosaic based on current knowledge describing miRNA-mediated regulation of genes, enzymes, or transcription factors involved in this biosynthesis process. At the same time, we would like to underline the fact that further research is necessary to improve our understanding of the miRNAs regulating plant lignan biosynthesis by exploitation of current approaches for functional identification of miRNAs.

## 1. Introduction

Phenylpropanoid metabolites biosynthesis is a complex network producing various important secondary metabolites, including lignans, and its regulatory mechanism is important for plant growth, development, and (a)biotic stress protection [1]. The most recent studies are focused on unraveling the miRNAs regulatory potential in the biosynthetic pathways of major secondary metabolites such as alkaloids, terpenoids, and flavonoids [1,2,3,4]. Lignans are polyphenolic compounds that are widely distributed in plant species. They represent one of the main components of plant cell walls [5] and show a variety of biological functions, including health-promoting effects. Despite their common biochemical basis, they possess wide structural variability. It is believed that there are up to 30,000 variants of lignans, of which only about 6000 are currently known [6]. Their strong biological activity encourages biochemists to create synthetic substitutes or incorporate them into drugs [7]. The wide spectrum of their structural and functional variability made it possible to identify certain types of lignans as good candidates for natural agrochemicals with the potential to inhibit seed germination and plant growth [5]. 

There are two main roles attributed to the lignans: plant defense and antioxidant activity [8]. The lignan content and compositions are significantly dependent on genetic and environmental background, including some other factors such as the maturity stage of the seeds and fruits [8,9]. Environmental factors can affect secondary metabolites biosynthesis by modulation of gene expression through miRNAs and transcription factors. The list of miRNAs and related transcription factors involved in flavonoid biosynthesis mediated by environmental factors is displayed in the following study [10].

Lignans biosynthesis is spatially and temporally dependent [11,12,13,14] as well as species and genotype specific [15,16]. 

MicroRNAs (miRNAs), as non-coding RNA molecules that bind to highly complementary sites in target mRNAs, play a key role in the post-transcriptional regulation of genes. They are highly conserved and have formed part of the genomes of viruses, plants, animals, and humans [17,18]. In addition to highly conserved miRNAs, less conserved miRNAs are also found in plants and interfere in several aspects of plant regulation such as response to biotic and abiotic stresses, regulation of developmental processes—leaf morphogenesis, physiology of cellular structures, alteration of vegetative phase, and flowering time [19,20].

Phenylpropanoids represent the largest group of plant secondary metabolites, involved in many biological and developmental processes, which at the same time points to their importance and meaning for plants. It is known that miRNAs also participate in phenylpropanoid biosynthesis by targeting transcription factors [1]. Therefore, the knowledge of the miRNA-based regulatory mechanisms of their biosynthesis is extremely important [2,3]. Currently, there is an effort to increase the production of secondary metabolites, which are produced by medicinal plants in small quantities and are used as insecticides, dyes, drugs, and toxins in agriculture, medicine, and industry. One of the ways how to increase their production is exploitation of RNA technologies that target small non-coding RNAs, which requires the knowledge of miRNAs involved in secondary metabolite biosynthesis [21].

While the genetic regulation of lignans biosynthesis is handled at a very good level, the direct links between this biosynthesis and miRNA-based regulation do not reach such a level of complexity [22,23]. In the review, we focused on the individual steps of lignan biosynthesis and searched for available information on the molecular regulation of these processes. Given the need for a deeper knowledge of the stress adaptive, nutritional, and medicinal potential of lignans, we would like to contribute to the understanding of the regulatory role of miRNAs in these perfect metabolic mechanisms and provide an overview of current approaches for functional miRNA assays in secondary metabolites biosynthesis.

## 2. Plant Lignan Biosynthesis and Tissue Compartmentation

**Figure 1:**
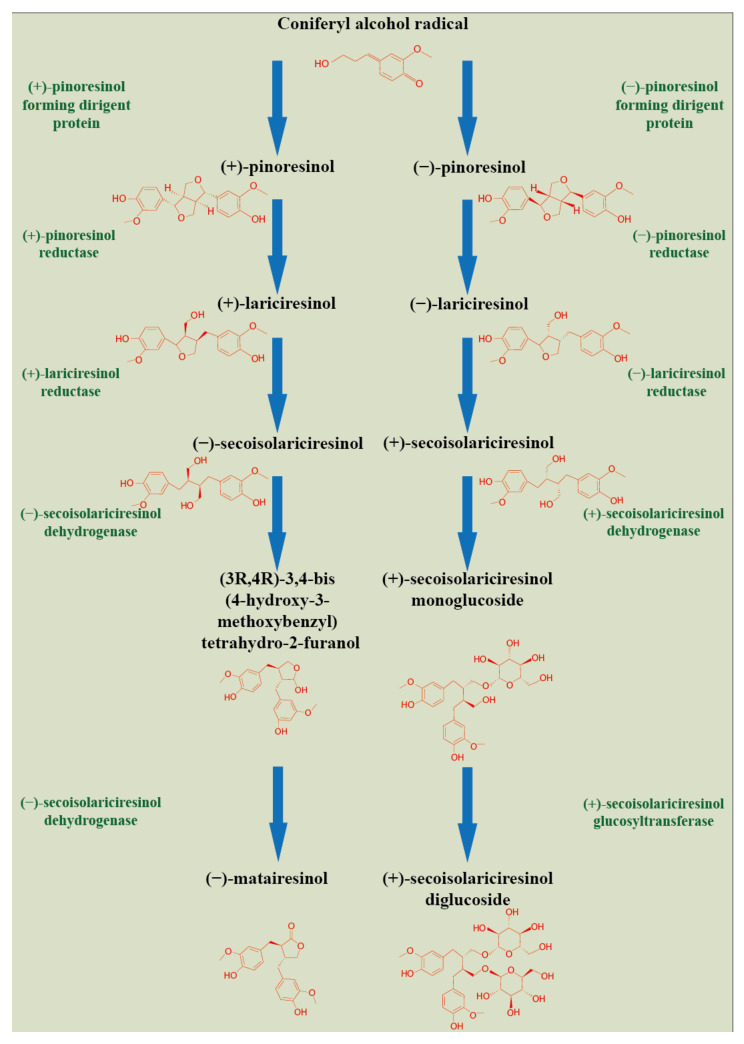
Plant lignans biosynthesis. (Harenčár, modified based on [26]).

Lignans, belonging to the group of phenylpropane derivatives (phenylpropanoids), are biosynthesized in the cell cytoplasm through the action of phenylalanine ammonia lyase (PAL) and pinoresinol-lariciresinol reductase (PRL), which catalyze the lignans biosynthesis [5]. They are synthesized by the shikimate pathway using the aromatic amino acid phenylalanine [24]. The product of phenylalanine deamination is cinnamic acid, which is further reduced by the ester of coenzyme A to an intermediate aldehyde. Subsequent hydroxylation by the P450 enzyme leads to the formation of hydroxycinnamic acid (p-coumaric acid) and/or its polyhydroxyl analogs. Their methylation by O-methyltransferase results in monomeric phenylpropanoid subunits—cinnamic acid derivatives, with subsequent reduction by the NADPH enzyme to alcohol. These compounds—hydroxycinnamic acid derivatives, aldehydes, and alcohols—represent monomeric units in the biosynthesis of lignans or neolignans. Dimerizations of these monomers are mediated by laccases, or peroxidases, followed by postdimerization transformations by methylation and/or hydroxylation processes [24,25]. A schematic of the synthesis of lignans is illustrated in Figure 1.

**Figure 2:**
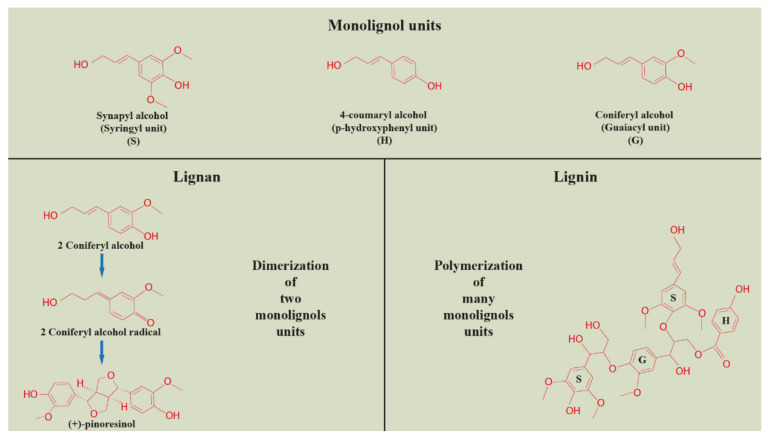
Dimerization and polymerization of monolignols units in the process of lignan and lignin biosynthesis, respectively (Harenčár, modified based on [26]).

As was already mentioned, more information has been available on the action of miRNAs in lignin biosynthesis. Since the biosynthesis of lignan and lignin monolignols shares common steps, it is possible to apply this knowledge to both, including subsequent dimerization (lignans) or polymerization (lignin) of monolignols [27]. Both lignans and lignins are polyphenols derived from the shikimate pathway, sharing common phenylpropanoid precursors—monolignols. Subsequently, the biosynthesis of lignans and lignins branches off, either to the process of dimerization (lignans) or polymerization (lignin) of monolignols (Figure 2). The biosynthesis pathway of these polyphenols is also sharing common enzymes, including the monolignol dimerization or polymerization process, where laccases and peroxidases are actively present [28,29,30]. The regulation of monolignols production and polymerization, as well as lignan production, includes NAC (NAM, ATAF1/2, CUC2) and MYB transcription factors [31].

The biosynthetic pathway of lignin is divided into two branches: the phenylpropanoid pathway from phenylalanine to hydroxycinnamic acid and the monolignol pathway from hydroxycinnamic acid to monomeric phenylpropanoid subunits (monolignols) [32]. The structure of lignin is a heteropolymer composed of three hydroxycinnamyl alcohol monomers, coniferyl alcohol, p-coumaryl alcohol, and sinapyl alcohol, which differ in their degree of methoxylation [33]. These monolignols produce guaiacyl (G), p-hydroxyphenyl (H), and syringyl (S) phenylpropanoid units when incorporated into the lignin polymer [27]. As with lignans, the amount and composition of lignin vary depending on the plant species, developmental phase, tissue type, and environmental conditions [34]. The lignins of dicotyledonous plants are generally composed of a combination of G and S (with less H) units in comparison to monocotyledonous plants, where these phenylpropanoid units are almost evenly represented [33]. 

In lignin biosynthesis, 15 genes were identified, and the binding sites for lignification-associated transcription factors (AC-I, AC-II, AC-III, and AC-IV) were identified in their promoter regions [33]. These factors bind to the promoter regions through *cis* regulatory elements related to hormone response (to ABA, salicylic acid, and methyl jasmonate), environmental stress, and development stage [33]. The analysis also indicated that the genes involved in monolignol biosynthesis may represent a putative target for members of the following miRNA families miR160, miR164, miR166, miR167, miR169, miR171, miR 5384, and miR6223 [33]. Several miRNA families such as miR156, miR164, and miR397 are involved in the lignin metabolism, content, and composition [35]. The regulatory network of microRNA-Long Non-coding RNA-transcription factors showed that specifically the regulation patterns of lnc6873-MYB2, lnc4458-MYB330, and lnc6437-MYB308 may be involved in lignin biosynthesis [1]. The crucial role of miR397 in the direct regulation of 12 laccase genes and 4 peroxidase genes involved in lignin polymerization was verified, including MYB (MYB021, MYB52) and NAC (SND1-A2, VND6-C2) transcription factors, directly activated by these genes [36]. The specific features of lignans and lignin are shown in Table 1.

The enantiomeric composition of some lignans was already determined, for example, in trees, medicinal plants, and some plant foods (linseeds, sesame seeds). It is known that etantiomers of the same type of lignan may show different biological properties and elicit different biological effects [9]. Stereochemical characterization of lignans showed varied etantiomeric composition in the whole berries compared to the seeds [9].

The composition of polyphenols in the lignan macromolecule varies during the developmental stages of the flax seed [8]. Monolignol glucosides accumulated at the early stages of seed development in a free form, whereas SDG was bounded inside the lignan macromolecule and accumulated mainly in the later stages. One of the explanations for the lignan macromolecule formation is their role in lipid protection against oxidation. The oilseed lignans are concentrated in the hull and those of berries or fruits in the seeds or kernels [9]. However, in the case of sesame seeds, some of the lignans are concentrated in the hull (lariciresinol, cyclolariciresinol), whereas others (sesamin) are evenly distributed in the seed. The lignan—podophyllotoxin (PTOX) shows phytotoxicity; therefore, it must be stored in the vacuoles as glucosides of the PTOX-producing plant cells [37]. Lignans have been identified except seeds in flowering aerial parts [38], leaves, roots [39], fruits and vegetables [40], and wooden parts [41]. The immunohistochemical labeling allowed to localize the lignans mainly in the secondary wall of the sclerite cells of the outer integument of the seed, and very light labeling was also observed in cytoplasmic inclusions of the endosperm [42].

## 3. Genetic Regulation of Plant Lignan Biosynthesis

Dirigent (DIR) proteins are considered mediators of lignans and their biosynthesis in the plant response to abiotic stress [43]. The expression of selected *DIR* genes has been shown to be involved in the formation of (−)-pinoresinol in the seed coat and (+)-pinoresinol in vegetative organs [44]. The synthesis of lignans involves the dimerization of two coniferyl alcohols. This step, directed by a dirigent protein (DIR), leads to the formation of pinoresinol (PINO) [44]. Consequently, the pinoresinol is converted to lariciresinol (LARI) and then to secoisolariciresinol (SECO) by the pinoresinol-lariciresinol reductase (PLR) [28,45]. The conversion of the (−)-pinoresinol to (−)-lariciresinol is catalyzed by pinoresinol reductase (PrR), which is coded by two genes, *PrR1* and *PrR2*. The promoter of the *PrR1* gene is regulated by transcription factors SND1 and MYB46 [46]. The transcription activity of the key gene in lignan synthesis, pinoresinol-lariciresinol reductase (*PLR*), was studied by RT-PCR and promoter-reporter transgenesis during the flax seed development. *LuPLR* gene was expressed in the seed coat, and consequently, the synthesis of PLR enzyme in mature seeds confirmed its involvement in SDG synthesis [47]. To better understand the function of the PLR enzyme in lignan biosynthesis, the RNAi strategy using hairpin dsRNA structures was applied for functional analysis of the *LuPLR1* gene [48]. Transgenic RNAi plants with silenced *LuPLR1* gene failed to accumulate SDG. HPLC analysis of lignan and neolignane content displayed a dramatic decrease in SDG in transgenic seeds.

The glycosylation of secoisolariciresinol (SECO) into SDG (secoisolariciresinol diglucoside) is achieved by uridine glycosyltransferases (UGTs). It has been shown [49] that the *UGT74S1* gene is the only one able to glycosylate SECO into SDG despite the identification of two duplicated genes (*UGT74S4* and *UGT74S3*) closely related to *UGT74S1* but incapable glycosylation.

Regulatory proteins known as transcription factors (TFs) are responsible for spatio-temporal synthesis and accumulation of secondary metabolites. TFs regulate the expression of target genes by binding to promoter-specific sequences, *cis* elements, in response to phytohormones and environmental conditions [50]. Investigation of spatio-temporal expression of *DIR* multigene family in flax genome revealed the function of specific *DIRs* in (−)-pinoresinol formation in seed-coats and (+)-pinoresinol in vegetative organs and/or specific responses to stress [44]. Phylogeny analysis grouped flax *DIRs* genes into six distinct clusters, where representatives of cluster DIR-a were connected to lignan biosynthesis, and those in clusters DIR-b/d and DIR-g have their putative function in lignin biosynthesis. A genome-wide analysis of the flax dirigent protein family records an extensive study by authors [44].

The enantiomeric composition of lignans is not only species-specific but also shows spatial and temporal specification. Similarly, pinoresinol-lariciresinol reductase (*PLR*) genes display spatio-temporal and organ-specific expression patterns [28,51]. Pinoresinol-lariciresinol reductases with opposite enantiospecificity determine the enantiomeric composition of lignans in different plant organs [51]. These enantiomers may represent the final products of lignans biosynthesis or are precursors for the synthesis of other groups of lignans [28]. Detailed characteristics of pinoresinol-lariciresinol reductases are provided in this review [28]. This suggests that the regulation of *PRL*s genes expression is crucial for lignans synthesis, and this statement is supported by the modification of *PRL*s gene expression patterns under in vitro induced stress conditions [47]. Stimulated expression of several lignan biosynthesis genes (*DIR*, *PLR*, and *UGT*) was observed under the elicitation effect of chitosan in addition to a significant increase in biomass of flax cell cultures [52].

## 4. The Involvement of miRNA in Plant Lignan Biosynthesis

Current trends in understanding the regulatory mechanisms of secondary metabolite biosynthesis point to a transcriptional level of regulation mediated by transcription factors, as well as to a post-transcriptional level of regulation with the involvement of interfering RNA molecules (miRNAs) [50,53]. There are several miRNA-mediated or miRNA-targeted modules of secondary metabolites regulation: miRNA-targeted biosynthesis-related genes, miRNA-targeted transcription factors (TFs), and miRNA-targeted noncoding RNAs (ncRNAs) [23]. The current observations pointed out that not only do miRNAs directly regulate lncRNAs but that they also indirectly regulate TFs through targeting of lncRNAs [1]. Regulatory machinery of gene expression is triggered through a combinatorial action of miRNAs and TFs [1,54]; additionally, they control the expression of each other. Most of the miRNA targets are transcription factors (TFs) which regulate plant growth, development, secondary metabolites biosynthesis, and stress tolerance [22]. The list of miRNA-targeted transcription factors involved in regulating flavonoid biosynthesis in plants is stated in the current study [55].

**Figure 3:**
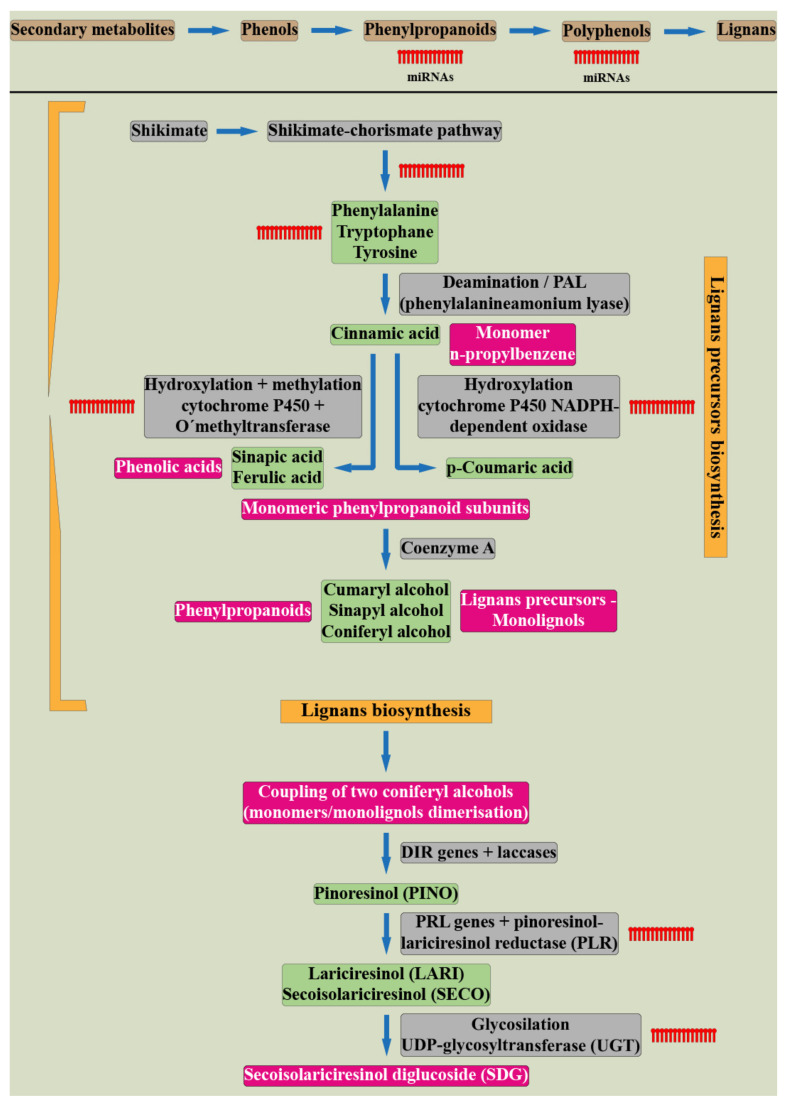
The schematic representation of the lignans biosynthesis indicating the known points of the pathway in which the miRNAs participate (Ražná, Harenčár, elaborated based on [55,56,57,58,59,60,61,62,63,64,65,66,67,68,69,70,71,72,73].

The phenylpropanoid pathway and the role of miRNAs in flavonoid biosynthesis is one of the most extensively studied [52,56]; however, the miRNA-based regulation of lignans is not characterized sufficiently. The schematic representation of the shikimate pathway leading to lignans precursors production followed by lignans biosynthesis and their interactions with known miRNAs is depicted in Figure 3.

A recent study [1] identified 80 differentially expressed genes (*DEGs*) involved in the phenylpropanoid biosynthesis pathway, which belong to 14 gene families (*PAL*, phenylalanine ammonia-lyase; *4CL*, 4-coumarate-CoA ligase; *C4H*, cannamate-4-hydroxylase; *CCR*, cinnamoyl-CoA reductase; *F5H*, ferulate-5-hydroxylase; *COMT*, caffeic acid 3-O-methyltransferase; *CCoAOMT*, caffeoyl-CoA O-methyltransferase; *HCT*, hydroxyl cinnamoyl transferase; *CAD*, cinnamyl-alcohol dehydrogenase; *POD*, peroxidase; *C3H*, p-coumaroyl ester 3-hydroxylase; *CALDH*, coniferyl-aldehyde dehydrogenase; *β-G*, beta-glucosidase; and *CGT*, coniferyl-alcohol glucosyltransferase. These *DEGs* were regulated by 23 miRNAs, whereas five *4CL* genes were targeted by miR393a-5p, four *C4H* genes by Novel325/353/418/424 miRNAs, and *C3H* was targeted by Novel234 miRNA, which points to a significant effect of *4CL*, *C4H*, and *C3H* genes on phenylpropanoid metabolism. In addition to this, the study [1] revealed that miRNAs and long non-coding RNAs (lncRNAs) indirectly regulate phenylpropanoid biosynthesis by targeting MYBs transcription factors, which act as repressors (MYB4, MYB39, MYB44, and MYB308) or activators (MYB2, MYB5, MYB12, MYB330, and MYB340) of phenylpropanoid metabolite pathway. This miRNAs-lncRNas-TFs co-regulation pattern contributes to tissue-specific variability of phenylpropanoid metabolites biosynthesis and is essential for regulating plant organ development [1,57].

Lignans, together with other polyphenolic metabolites (which are categorized into flavonoids), are synthesized by a central phenylpropanoid pathway, sharing common enzymes and substrates. Several miRNAs were recognized to regulate flavonoid biosynthesis by targeting the mRNAs encoding key enzymes [58] or transcription factors of the phenylpropanoid pathway [55]. O-methyltransferase in phenylalanine metabolism is targeted by miR1438. Activation of 4-coumarate and 4-hydroxycinnamates to the respective thiol esters is regulated by miR172i. Activated thiol esters are then used as a precursor of monolignols.

Cytochromes P450 (CYPs, CYP450, and P450) are heme-containing enzymes. They are known as monooxygenases catalyzing oxygenation/hydroxylation reactions in the biosynthesis of many secondary metabolites [59,60]. The microarray analysis of 326 *CYP* genes in the rice genome displayed tissue-specific and zone-specific expression patterns. At the same time, differential expression of several *CYP* genes was observed in response to drought stress. It has been confirmed that the expression of *CYP* genes can be post-transcriptionally regulated by miRNAs families, such as miR440, miR531, miR812, and by species-specific families miR1848, miR5523 (rice genome) [59]. It has been elucidated that *CYPs* involved in the phenylpropanoid pathway are primarily regulated and targeted by miR413 [60]. A Cytochrome P450 has been found to be a target for miR5035, miR2275d [61], and for lus-miR168b [62].

Phenylalanine ammonia-lyase (PAL) is the key enzyme of the phenylpropanoid pathway, participating in the precursor production of important secondary metabolites. PAL encoding genes are generally well studied [63]; however, the knowledge of the underlying regulatory mechanism of miRNAs involved in the *PAL* gene regulation does not reach the same level. Twelve common walnut *PAL* genes were targeted by several miRNAs belonging to miR172, miR399, miR408, miR477, miR830, and miR7729 families, pointing to tight regulation of *PAL* genes at the pre- and post-transcriptional level [63]. Inhibition of *PAL* gene expression by miR477 was observed as a response to pathogen infection [64]. The phenylalanine ammonia lyase (PAL) converting L-phenylalanine to ammonia and trans cinnamic acid is a target for miR1873 [65].

Laccases (LACs) belong to oxidase family enzymes (multicopper oxidoreductases). Due to containing four copper (Cu) ions, they can catalyze the oxidation of various aromatic and nonaromatic substrates, including oxidative polymerization of monolignols [66]. Laccase genes are responsible for the polymerization of monolignols, and post-transcriptional regulation of these genes by miRNAs was identified [29]. Almost 82% of soybean laccases are potential targets of miRNAs, such as miR397a/b, miR408d, and miR5671a, whereas maize laccase genes were potentially targeted by miR397a/b or miR528a/b. Expression analyses of soybean and maize laccase genes revealed tissue- and developmental-specific activity profile, including their specific response to abiotic and biotic stress factors [66]. Transcripts of six flax (*Linum usitatissimum* L.) laccase genes were targeted by lus-miR397 [67]. Almost 59% of identified laccase genes in *Populus trichocarpa* Torr. & Gray were predicted to be target of ptr-miR397a. Transgenics overexpressing in *Ptr-MIR397a* resulted in reduction of laccase genes expression as well as in lignin reduction, indicating that the biosynthesis of lignin is regulated by miRNA [68]. In the *P. trichocarpa* Torr. & Gray genome, three miR397 (miR397a, b, and c) were identified; however, only ptr-miR397a was sufficiently abundant to be detected [67].

The glycosylation of SECO into SDG is regulated by UDP-glycosyltransferases (UGTs, or uridine diphosphate glycosyltransferases). Flax *UGT74S1* gene coding UDP-glycosyltransferases was reported as the key enzyme controlling SECO glycosylation in flax [69,70], using SECO as substrate to form SDG.

As lignans and lignins are phenylpropanoid substances sharing common enzymes and substrates [53], some of the knowledge of miRNA-mediated lignins regulation might be implemented in understanding miRNA-mediated lignans biosynthesis. Transcripts belonging to the blue copper protein (BCP) family are targeted by miR408. The functions of these proteins include oxygenase and oxidase activities and monolignol coupling catalysed by laccases within the cell wall [71]. In Arabidopsis, miR408 has been shown to regulate laccase genes involved in cell wall lignification [72]. Together with another two miRNAs (miR397 and miR857), it has been shown to target the transcripts for the members of the lignin-related laccase copper protein family [72]. The *LIM1* gene, which may act as a transcriptional activator of lignin biosynthesis, is targeted by miR167h [73].

To obtain insight into the regulation of lignan biosynthesis during *Schisandra chinensis* (Turcz.) Baill. fruit development, long-read transcriptome sequencing was conducted. More than 21,800 assembled transcripts (or unigenes) were classified into 58 TF families, amongst which were identified as most abundant: bHLH, NAC, WRKY, MYB/−related, FAR1, C3H, B3, C2H2, ERF, GRAS, bZIP, HB-other, TALE, G2-like, YABBY, and Trihelix families [74]. Almost 13,000 unigenes were assigned to 20 primary pathways (according to KEGG pathway assignments), where the most represented were ‘metabolic pathways’ and ‘biosynthesis of secondary metabolites’. Subsequent analysis for transcripts related to ‘biosynthesis of secondary metabolites’ besides other pathways confirmed those related to ‘phenylpropanoid biosynthesis’ [74].

Several types of transcription factors have been implicated in the regulation of lignan biosynthesis. MYB (myeloblastosis) is a large family of proteins having different numbers of MYB domain repeats; for example, MYB-related or R2R3-MYB. MYB TF regulates, among others, the biosynthesis of secondary metabolites [9]. MYB-related transcription factors regulate flavonoid metabolism in plants and are incorporated in the regulation of phenylpropanoid metabolism [75]. MYB12 transcription factor belonging to the group of R2R3-MYB transcription factors represents a flavonol-specific regulator of phenylpropanoid biosynthesis [50,76] and is putatively targeted by miRNA858 [77]. The expression of several MYB transcription factors was downregulated by overexpression of miR858. Higher expression of MYBs in *Arabidopsis thaliana* (L.) Heynh. transgenic lines expressing artificial miRNA target mimics (*MIM858*) resulted in supported flavonoids synthesis at the cost of lignin synthesis [77].

The APETALA 2 (AP2)/ethylene-responsive factor (ERF) belongs to transcription factors (TFs) families, well-documented in plant responses to a wide range of biotic and abiotic stresses [78], in the control of primary and secondary metabolism and plant growth and development [79]. Positive regulation of plant lignans biosynthesis by APETALA2/ethylene response factor (AP2/ERF) was suggested. Knocking down the expression of li049, one novel AP2/ERF gene from *Isatis indigotica* Fort., caused a remarkable reduction of lignans/lignins contents [80]. AP2-domain-containing TF are also targets for lus-miR172e [81].

The involvement of a WRKY transcription factor in the regulation of lignans biosynthesis in flax was described. WRKY TFs bound to W box of *LuPLR1* (pinoresinol-lariciresinol reductase 1) gene promoter, incorporated in secoisolariciresinol synthesis. It was also pointed out that LuWRKY36 positively regulates the *LuPLR1* gene expression of lignans biosynthesis in response to biotic stress. More effective *LuPLR1* gene expression was associated with higher secoisolariciresinol accumulation in the hypersensitive reaction of the resistant flax cultivar [28].

The scientific community has devoted considerable attention to the identification and characterization of miRNAs involved in the regulation of several secondary metabolites’ biosynthesis (Table 2). The progress made in this area is provided by several studies: Refs. [23,58,82]—describing microRNA-mediated regulation in flavonoid, terpenoid, alkaloid, and other N-containing metabolites biosynthesis; ref. [55]—pointed out the significance of miRNAs in the enhancement of flavonoid biosynthesis; ref. [83]—characterizes lncRNA mediated gene expression in flavonoids and terpenoids production in *Citrus limon* (L.) Osbeck; ref. [84]—describing miRNA role in phenylpropanoid, terpenoid, alkaloid, chlorophyll, and carotenoid biosynthesis; ref. [85]—identifies the role of miRNAs in phenylpropanoids, terpenoids, and alkaloids biosynthesis in medicinal plants; ref. [86]—is focused on lncRNAs regulatory potential in *Populus trichocarpa* Torr. & Gray phenylpropanoid pathway; ref. [87]—identifies miRNAs in secondary metabolites of *Murraya koenigii* (Linn.) Spreng; ref. [88,89] —states transcription factors and miRNAs mediated flavonol biosynthesis in *Solanum tuberosum* L.; ref. [90]—identifies miRNAs involved in artemisinin biosynthesis in *Artemisia annua* L.; ref. [91]—introduces miRNA-based regulation of secondary metabolites biosynthesis with pharmaceutical applications in medicinal plants; ref. [92]—reveals miRNA effects on secondary metabolism in *Solanum tuberosum* L.; and ref. [93]—characterizes the role of miRNA858 in phenylpropanoid pathway.

## 5. Current Approaches for Functional Identification of miRNAs in Plant Metabolites Biosynthesis

Identification and characterization of miRNAs participating in the regulatory mechanism of plant secondary metabolites is an important research task for the promising and targeted use of secondary metabolites and their effective modulation.

**Figure 4:**
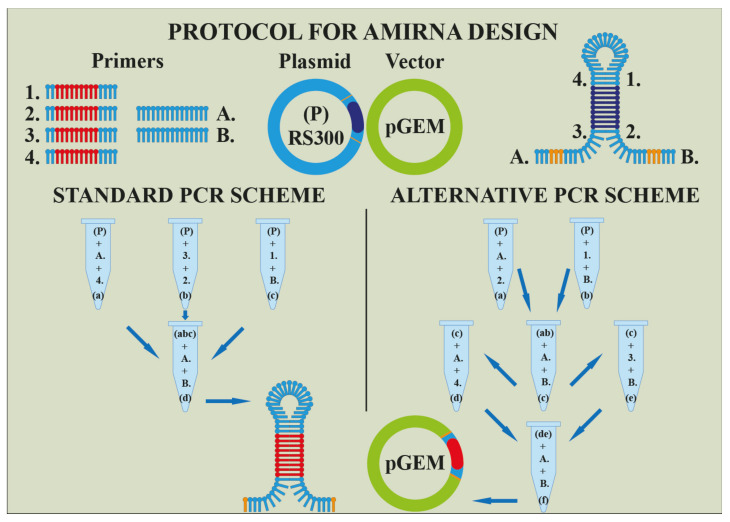
An illustration of projecting artificial MicroRNA or syn-tasiRNA, respectively (Harenčár, modified based on [112,113,114]). The artificial MicroRNA is made by a sequential adding of primers (1.; 2.; 3.; 4.; A.; B.;) to the main template (P) and its intermediate products ((a), (b), (c), respectively (d)) during individual PCR reactions. The final product ((d) or (f)) is transferred to the vector pGEM.

Many methodologies have evolved due to understanding microRNA silencing complex and their impact on plants organism. Within genetic engineering exist two more approaches besides the knock-outing of mentioned enzymes or factors. The first one lies in an over-expression of miRNAs and the second one in inhibition of miRNAs. Into the first category belongs technology AmiRNA or syn-tasiRNA (Figure 4). Based on a genomic construct composed of a cloning vector and primary microRNA with a stem-loop structure, a specific miRNA is over-expressed and silences genes effectively [104,105].

**Figure 5:**
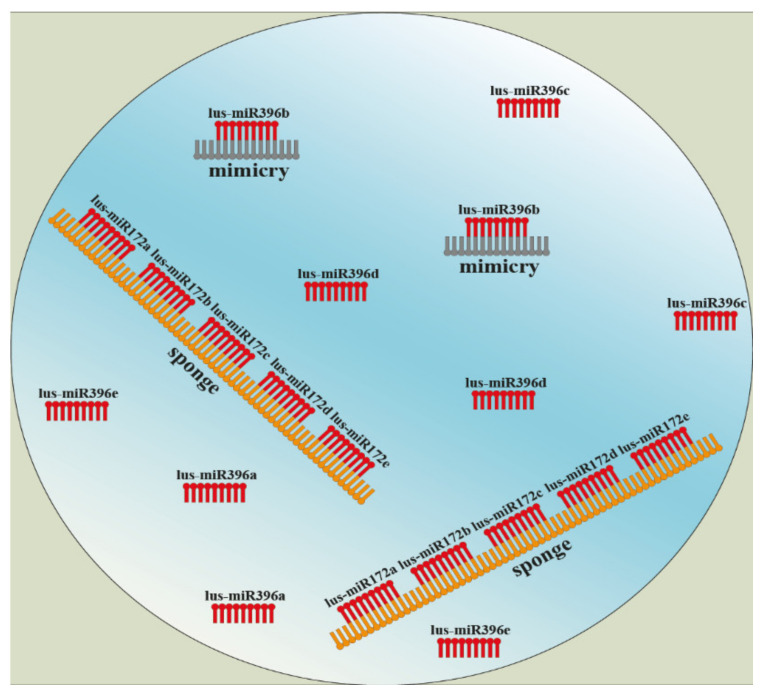
Design and main principle of microRNA sponge and noncoding endogenous target mimicry (eTM) technology. (Harenčár, modified based on [111,115,116]).

Among techniques that use a silencing of microRNAs are noncoding endogenous target mimicry (eTM), short tandem target mimic (STTM), or miRNA sponges. Mimicries are endogenous non-coding or low-coding sequences with different lengths. They are part of long non-coding RNA (lncRNA), and their epigenetic function is inhibition of microRNAs by mutual binding, which creates a mismatch loop in the middle of specific miRNA. Due to this incomplementarity, the miRNA is not able to cleavage and silence other mRNAs [106,107]. Based on this knowledge, the short tandem target mimic (STTM) technology was developed to inhibition of endogenous microRNAs. The main point again lies in a design of a specific STTM construct and a genetic transformation [108,109,110]. A MicroRNA sponge is an expanded methodology where a construct usually contains a whole family of microRNA, e.g., MIR-172a…MIR-172p (Figure 5) [111].

**Figure 6:**
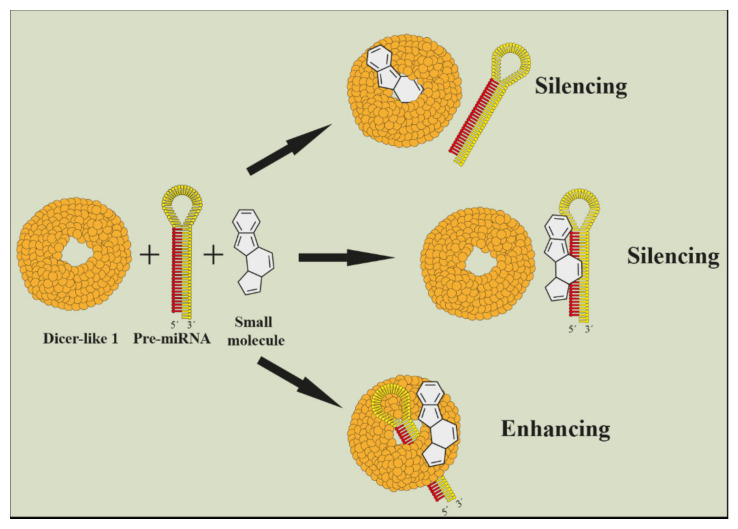
Illustration of small molecule as enhancer or inhibitor of a microRNA maturation (Harenčár, modified based on [117,118,119,120,121,122,123,124]).

For the purpose of inhibiting microRNA, it was found that small molecules can also stop a miRNA maturing by binding onto primary miRNA and preventing it from Dicer cleavage [117,118,119,120,121]. This technology is also commercially available [122,123]. Small molecules can be used as well as enhancers of microRNA cleavage [124]. Scientists identified several plant viruses that caused abnormalities by a specific microRNA upregulation [125] (Figure 6). Imaging of gene silencing in real time is also interesting [126,127].

In silico approach represents one of the fastest developing ways how to understand the main role and big potential of microRNAs not only in plant secondary metabolites synthesis. Currently, as a primary source of information serves databases, and among the most famous belongs miRbase [118]. Plenty of software and algorithms use these data and process them according to established criteria and intention. Because microRNAs complementary pairing with their target gene sequences, algorithms used to work with computations based on permitted match and mismatch eventually on free energy [128]. Scientists usually make their algorithms accessible in the form of command lines written by a specific programming language executable in the power shell of the operating system, for example, Novomir [129]. Another has a user interface; however, they use a processor of personal computers such as p-TAREF [130], or it exists online web servers with a shared supercomputer, machine learning, adjustable criteria, and database of microRNA sequences. One of them is psRNATarget [131,132]. Many reviews and web pages follow up tools for microRNA prediction [128,133,134]. Due to the designing of constructs and genetic engineering with microRNAs, bioinformatic models were devised that help to succeed in transgenesis. Some techniques mentioned above (amirna, sponge, SSTM, syn-tasiRNA, etc.) have their own in silico design servers as well, e.g., [135,136,137,138]. RNA-induced silencing complex is quite a novel part of epigenetics applicable in medicine, nutritional science, metabolomics, and genomics and, in combination with bioinformatics, represents a big scientific potential for the future. For this reason, methods, techniques, and algorithms will be on the increase [139,140,141,142,143].

## 6. Conclusions

Due to the biological importance of secondary metabolites, understanding the regulatory mechanisms of their biosynthesis is crucial [36]. Plant lignans hold significant biological potential, not only in terms of nutritional and medicinal use, but also in terms of plant adaptive potential to environmental stress. Lignans biosynthesis pathway is known in detail; however, the knowledge of the involvement of miRNAs in regulatory processes of the biosynthesis is partial. The current understanding is limited to identification of miRNA-mediated regulation or to characterization of involved transcription factors of phenylpropanoid pathway and its individual groups of metabolites (alkaloids, flavonoids, and terpenoids). The review is focused on the individual steps of lignans biosynthesis and matching identified and characterized miRNAs and transcription factors (as primary target sequences for miRNAs) to make a mosaic of miRNA-modulated regulation of plant lignans biosynthesis. Whereas understanding the role of miRNAs in the regulation of plant lignan biosynthesis represents an important research task, the review presents several current approaches to miRNA functional analysis. We believe that the summarized information will create a knowledge platform for further screening of miRNA functions and thus contribute to a more detailed understanding of miRNA-based regulation of lignans biosynthesis.

## Figures and Tables

**Table 1:** Different properties of plant lignans and lignins.

Lignans	Lignins
Dimeric	Polymeric
Stereochemical diversity	Optically inactive
A variety of structural motifs	Different proportions of three types of monolignols within the lignin fractions
Species-specific properties of individual types of lignans	Species-specific proportionality of individual monolignols
Spatially and temporally specific accumulation	Spatially and temporally manner of lignification
Antioxidant, antibacterial, antiviral, fungicidal, insecticidal, estrogenic, antiestrogenic, anticarcinogenic properties	Mechanical strength of plant tissues, hydrophobicity, defense against pests and pathogens

**Table 2:** MiRNA and transcription factors involved in the phenylpropanoid biosynthesis regulation with focus on lignin/lignans target biosynthesis (in italics), elaborated based on references [1,2,3,4,31,33,35,36,46,57,68,94,95,96,97,98,99,100,101,102,103].

miRNAs	Transcription Factors
*miR156*; miR157; miR159; miR159e; *miR160*; *miR164*; *miR166*; miR166i; *miR167*; *miR167h*; *miR168b*; *miR169*; *miR171*; miR172; *miR172i*; *miR172e*; *miR393a-5p*; *miR396b*; *miR396e*; *miR397*; *miR397a/b*; *miR399*; *miR408*; *miR408d*; *miR413*; *miR440*; *miR477*; *miR528a/b*; miR530; *miR531*; *miR812*; miR829.1; *miR830*; miR845; *miR850*; *miR857*; *miR858*; *miR858a*; miR858b; miR894; miR530; *miR1536*; *miR1438*; *miR1873*; *miR2275d*; miR2891; miR3627; miR4376; *miR4391*; miR4995; *miR5035*; miR5298b; *miR5384*; miR5532; *miR5671a*; miR6194; *miR6223*; miR7695; *miR7729*; miR8154; *miR9567*; miR9662	*MYB family* (MYB1; *MYB*2; *MYB4*; *MYB5*; *MYB11*; *MYB12*; *MYB021*; MYB32; *MYB39*; *MYB44*; *MYB46*; *MYB52*; *MYB58*; *MYB63*; MYB75; *MYB88*; *MYB 111*; *MYB124*; *MYB 308*; *MYB330*; *MYB340*; *R2R3-MYB*); *AP2/ERF*; *NAC family (NAM; ATAF1/2; CUC2; SND1-A2; VND6-C2)*; SPL9; bHLH; TCP3; PAP1; WDR; *WRKY2*; *WRKY 36*; *LIM*

WDR—WD40 repeat proteins also termed TTG1—Transparent testa glabrous1; bHLH—basic helix-loop-helix; AP2/ERF—APETALA2/ethylene response factor; WRKY—zinc finger regulatory proteins; MYB—v-myb avian myeloblastosis viral oncogene homolog; PAP1—production of anthocyanin pigments; SPL—Squamosa promoter binding protein-like9; TCP—class II CIN-TCP protein TCP3; NAC—NAC domain-containing protein; VND6—vascular-related NAC-domain 6.

## Data Availability

Not applicable.
